# A Rare Case of a Life-Threatening Massive Upper Gastrointestinal Bleed and Airway Obstruction in a Patient With a Megaesophagus Secondary to Longstanding Achalasia

**DOI:** 10.7759/cureus.13204

**Published:** 2021-02-07

**Authors:** Raghav Bassi, Yasir Saeed

**Affiliations:** 1 Internal Medicine, Lincoln Medical and Mental Health Center, New York, USA

**Keywords:** pressure ulcer, severe achalasia, megaesophagus, upper gastro-intestinal bleed, partial airway obstruction, clinical case report

## Abstract

Achalasia is a relatively rare motor disorder characterized by esophageal aperistalsis and incomplete relaxation of the lower esophageal sphincter. In only 10% of patients, untreated or poorly managed achalasia can progress to esophageal dilation and eventual loss of total functionality resulting in a characteristic sigmoid dolichomegaesopahagus. In extremely rare instances, this sigmoid dolichomegaesopahagus can present clinically as acute airway obstruction or a fatal, life-threatening hemorrhage requiring immediate intervention. We present the case of a 65-year-old female with a past medical history of long-standing achalasia who had complaints of shortness of breath, chest pain, and two episodes of life-threatening hematemesis requiring a blood transfusion. An angiography illustrated significant distention of the esophagus occupying most of the right hemithorax and non-specific intraluminal fluid with a small amount of gas. Emergent esophagogastroduodenoscopy showed fibrosis and necrosis of the esophageal mucosa with food debris, suggesting that the bleeding was likely coming from an ulcer caused by pressure necrosis. The patient was hemodynamically unstable after the procedure and was transferred to another facility the next day for an esophagectomy. Patients with achalasia have an increased susceptibility to develop pressure ulcers due to increased shear force on the esophageal wall, increased moisture of the esophageal wall from prolonged contact of food boluses, and underlying malnutrition and weight loss from the indigestion of food causing atrophy of the mucosal barriers. The management of these ulcers is to treat and manage the underlying cause. Although there are no curative treatments for achalasia, symptomatic relief through both surgical and medical therapies are the mainstay of management, with an esophagectomy reserved for refractory cases or in patients who develop end-stage complications.

## Introduction

Achalasia is an idiopathic primary motor disorder characterized by esophageal aperistalsis and incomplete relaxation of the lower esophageal sphincter (LES) [1-3]. The underlying pathophysiology is attributed to a functional loss of the myenteric plexus of Auerbach, resulting in an impairment of the inhibitory postganglionic neurons in the distal esophagus and LES [4-6]. The inability of the LES to relax upon swallowing causes progressive dysphagia for both solids and liquids with accompanied retrosternal chest pain from the regurgitation of food debris and fluids, along with halitosis [1, 4, 7-8].

Achalasia is a relatively rare disorder with an incidence rate of 1 in 100,000 and a prevalence of 1 in 10,000 in the United States with an equal distribution in males and females [2, 6]. Clinically, the diagnosis of achalasia is often delayed because of its similar presentation with other more prevalent diseases such as gastroesophageal reflux disease (GERD) [6-7]. Untreated or poorly managed achalasia can have serious consequences such as progressive esophageal dilation, elongation, tortuosity, and the total loss of functionality resulting in a characteristic “sigmoid dolichomegaesopahagus” in 10% of patients [9-10]. Infrequently, sigmoid dolichomegaesopahagus can cause an acute airway obstruction from the regurgitation of food and present clinically as asthma, pneumonia, or a lung abscess requiring urgent treatment [4, 11].

Furthermore, in extremely rare situations, long-standing primary achalasia can also result in fatal esophageal hemorrhage due to underlying mucosal irritation and ulcer formation [2, 8, 12]. To our knowledge, the etiology of upper gastrointestinal (GI) bleeds in achalasia were due to cytomegalovirus (CMV) esophagitis, esophageal varices, excessive ingestion of tannins, esophagopulmonary fistula, non-Hodgkin esophageal lymphoma, and excessive aspirin ingestion [2, 4-5, 12-13]. We hope to further contribute to the current understanding of this topic by demonstrating a rare case of a patient with achalasia mediated megaesophagus who presented with acute respiratory obstruction and life-threatening esophageal bleeding from underlying pressure ulcers.

This article was previously presented as a poster presentation at the 2020 Annual American College of Gastroenterology Meeting on October 26, 2020 (poster: Saeed Y, et al. Life-Threatening Massive Upper Gastrointestinal (GI) Bleed and Airway Obstruction in a Patient With Megaesophagus From Longstanding Achalasia).

## Case presentation

A 65-year-old female with a past medical history of achalasia presented to the emergency department (ED) with complaints of worsening shortness of breath, nausea, non-bilious hematemesis with some regurgitated food and water, and chest pain over the past day. Her symptoms were accompanied by sweating, fatigue, and intermittent dysphagia for both solid foods and liquids. Although she declined any treatment, she continued to live a relatively normal life through lifestyle interventions consisting of a pureed diet, carbonated beverages to aid in digestion, eating smaller and more frequent meals, and indulging in specific maneuvers such as lifting her neck and shoulders back to further enhance esophageal emptying. The patient denied the regular use of tannins, NSAIDs or aspirin, immunosuppressants, and any unintentional weight loss, melena, and hoarseness of her voice.

Her vitals in the ED were significant for a temperature of 36.1°C, hypotension of 106/60 mmHg with a tachycardia of 110 beats/minute, respiratory rate of 14 breaths/min, and oxygen saturation of 96% on room air. Her physical exam was pertinent for pale conjunctiva and nail beds with a decreased capillary refill time. Her complete blood count (CBC) showed normocytic anemia with a hemoglobin of 9.5 g/dL, which was significantly lower than her baseline of 12 g/dL, a hematocrit of 23.6%, and leukopenia of 4.59x103/mL. The patient’s basic metabolic profile was unremarkable except for a slight acute kidney impairment with a creatinine of 0.88 and blood urea nitrogen (BUN) of 28.0, likely due to her hypotension. She was then given three liters of intravenous fluids, 40mg of Protonix®, and 10mg of Reglan® with mild symptomatic relief. 

**Figure 1:**
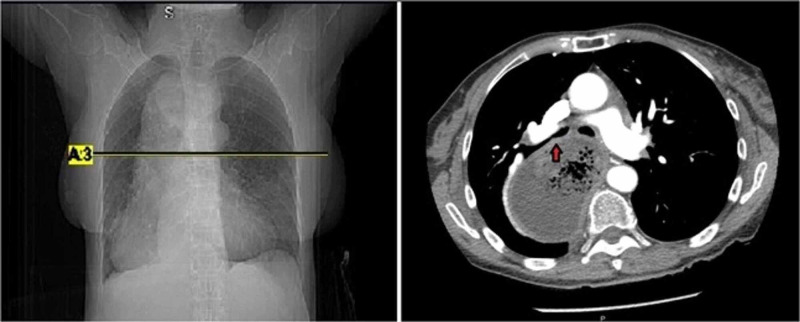
Computed tomography angiogram of the chest with an axial view (left) and corresponding coronal section (right) displaying a significantly dilated megaesophagus occupying most of the right hemithorax There is also some intraluminal fluid with a small amount of gas partially obstructing the right main bronchi (arrow).

**Figure 2:**
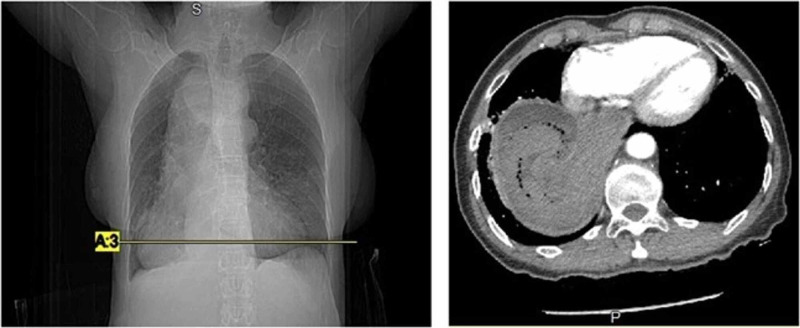
Computed tomography angiogram of the chest at a lower level with an axial view (left) and the corresponding coronal section (right) further showing a significantly dilated esophagus with small amounts of intraluminal fluid and gas

While she was being escorted for radiological testing, she experienced a second large episode of bright red hematemesis accompanied by severe dizziness and fatigue. A repeat CBC showed her hemoglobin dropping to 7.2 g/dL, and a blood transfusion was then given. A chest X-ray showed mediastinal widening with a dilated megaesophagus occupying most of the right hemithorax. In addition, CT angiography (CTA) was done to rule out aortic dissection, and it illustrated significant distention of the esophagus with non-specific intraluminal fluid with a small amount of gas (Figures 1-2). Luminal narrowing at the level of the esophagogastric junction was also evident. In addition, moderate ground glass opacifications were seen in the inferior right upper lobe, right middle lobe, right lower lobe, and left lower lobe suggesting possible atelectasis or aspiration with a dilated right main bronchus suggesting obstruction.

**Figure 3:**
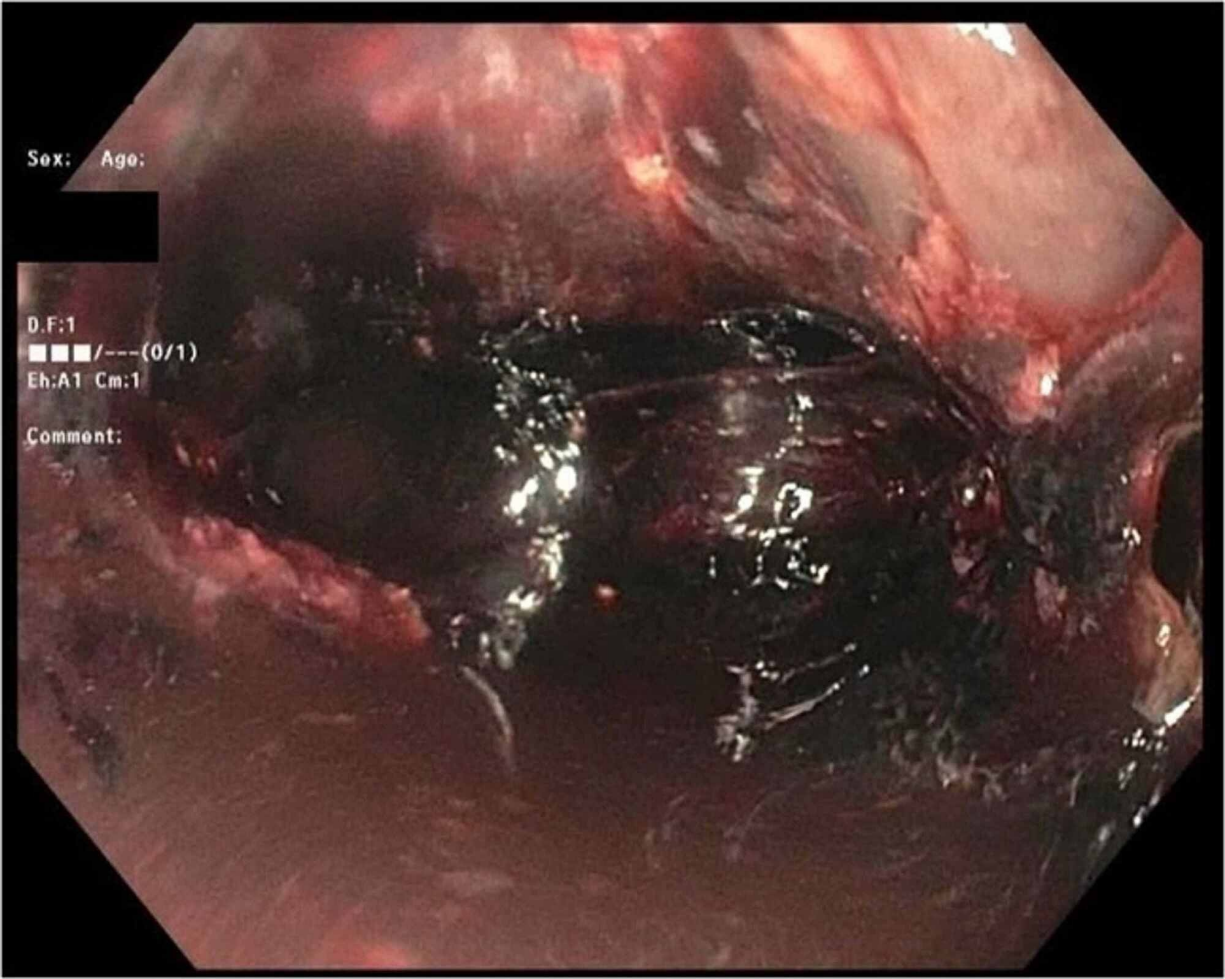
Esophagogastroduodenoscopy showing the esophageal mucosa with fibrosis and necrosis In addition, dark red blood was seen throughout, preventing adequate visualization of the bleeding source.

An emergent esophagogastroduodenoscopy (EGD) revealed areas of the esophageal mucosa containing fibrosis and necrosis with some food debris and diffuse dark red blood throughout, preventing adequate visualization of the bleeding source (Figure 3). These findings suggested that the bleeding was likely coming from an underlying ulcer caused by pressure necrosis from retained food. The endoscope was unable to pass through the gastroesophageal junction due to a severely dilated and aperistaltic esophagus. The patient was hemodynamically unstable after the procedure and was transferred to another facility the next day for an esophagectomy.

## Discussion

Achalasia is an esophageal motility disorder that is characterized by impaired relaxation of the LES during swallowing and aperistalsis in the lower two-thirds of the esophagus due to parasympathetic denervation [2, 7, 10]. Although the exact etiology of achalasia has not been elucidated yet, the neuronal degeneration has been associated with neurodegenerative or autoimmune processes as patients present with increased serum neural autoantibodies and with histopathological evidence of a T-lymphocyte mediated reaction [14]. Achalasia can be further categorized according to its etiology as either primary if it’s idiopathic, secondary if caused by parasitic infections like Trypanosoma cruzi as seen in Chagas disease, or in 2-4% of cases as pseudoachalasia, if associated with tumors, systemic neuromuscular or metabolic disease [6, 10]. Although there are many diagnostic and imaging modalities available, the management of achalasia involves early diagnosis to preserve both the myenteric plexus and esophageal function and minimize long-term consequences [3, 7]. When a patient presents with progressive dysphagia, the initial evaluation is to rule out GERD or mechanical obstructions caused by malignancies, strictures, webs, or rings through an EGD before focusing on esophageal motility disorders [3, 7, 10]. However, a normal EGD cannot rule out achalasia, as 40% of achalasia patients will have a normal endoscopy [15]. The gold standard for diagnosing achalasia is esophageal manometry, which demonstrates esophageal aperistalsis and increased LES pressure [1, 6-7]. Another commonly used diagnostic test is a barium esophagram, which shows a pathognomonic “birds beak” appearance of the distal esophagus with proximal esophageal dilation [7, 10].

Upper GI bleeding in achalasia is an extremely rare phenomenon and is associated with underlying mucosal ulcers caused by pill esophagitis, malignancies, or stasis ulcers [2, 4-5, 8]. Typically esophageal ulcers are formed due to the loss of the protective mucosal barrier caused by gastric acid refluxing through a weak LES as seen in GERD or repeated episodes of vomiting, as seen in bulimia nervosa [16]. Other causes also include infections in immunocompromised hosts such as candida species, CMV, and herpes simplex virus, or direct mucosal damage from the ingestion and stasis of caustic agents like tannins, nonsteroidal anti-inflammatory drugs (NSAIDs), bisphosphonates, and certain antibiotics [2, 5, 13, 16]. The long-term morbidity of achalasia is from aspiration pneumonia, spontaneous esophageal rupture, malnutrition, dysphagia, esophagitis, esophageal-tracheal fistula, and esophageal squamous cell carcinoma [2, 5]. As seen in the case presented above, this patient denied using caustic agents and likely presented with an upper GI bleed and respiratory complications resulting from retained food as observed throughout the EGD.

Pressure ulcers are unrelieved high-pressure areas due to friction or shearing forces that result in ischemia and tissue necrosis [16-18]. Although classically associated with post-surgical and elderly patients, they are also commonly found in younger patients with neurological disorders and have been shown to have a mortality rate of 22% in some studies [17]. Most pressure ulcers tend to occur over bony prominences such as the sacral, hip, heel, or elbow region; however, there have been case reports describing mucosal involvement such as the nasal cavity from nasal gastric tube placement in critically ill patients [18-19]. The underlying pathogenesis encompasses three direct causal factors: immobility, skin integrity, and poor perfusion [18]. Immobility can predispose patients to higher magnitudes of non-uniform stress, while moisture enhances the vulnerability of mucosal damage [16-18]. Mechanical loads that are distributed non-uniformly over a long period of time have a greater impact on tissue damage and integrity [17, 19]. In addition, general mucosal status or previous pressure ulcers can also increase the susceptibility of developing new pressure ulcers due to changes in the transportation of biomolecules [18]. Lastly, ischemia can decrease tissue viability and disrupt the normal healing processes resulting in tissue necrosis [17, 19].

When we consider these mechanisms in the underlying pathophysiology of achalasia, we can gain further insight into the increased susceptibility of pressure ulcer development in these patients. The hypercontractility of the distal esophagus results in the regurgitation of food, which can play a fundamental role in the non-uniform shearing or frictional forces seen in pressure ulcer formation. In addition, achalasia patients have a higher tendency to induce self-vomiting in order to provide symptomatic relief, further causing erosive damage to the protective esophageal mucosal layer and increasing the shearing force on the esophageal wall [6-7, 10]. Furthermore, retained food boluses have already undergone mastication and contain a high amount of saliva, which comprises 99% of water, with the remainder being amylase, lipase, bicarbonate, and lysozyme [20]. When these food boluses rich in salivary content make contact with the esophagus for prolonged periods due to incomplete LES relaxation, they can further increase the esophagus' moisture and predispose it to pressure ulcer formation. Lastly, patients with achalasia tend to present with underlying malnutrition and weight loss from food indigestion [9]. This malnutrition can lead to hypoalbuminemia and further hypotension, causing the atrophy of the mucosal barriers [17-18]. All of these factors working in concert in patients diagnosed with long-standing achalasia can increase the susceptibility of developing esophageal hemorrhage from pressure ulcers. 

There are currently no curative therapies available for achalasia. The intervention aims to reduce LES pressure in order to provide symptomatic relief to patients promoting food and gastric acid clearance and avoiding bacterial overgrowth [2, 7]. This can be accomplished surgically through pneumatic dilation or Heller’s myotomy and medically in poor surgical candidates through the use of calcium channel blockers, nitrates, sildenafil, or botulinum toxin [2-3, 7]. In patients with underlying esophageal ulcers, management aims to treat the underlying etiology with severe cases requiring nasogastric tube intubation, fluid resuscitation, histamine receptor-2 antagonists, or proton pump inhibitor pharmacotherapy, and antibiotic chemoprophylaxis [16]. Furthermore, cases refractory to medical management and patients who develop large episodic hematemesis from underlying ulcers secondary to end-stage complications such as megaesophagus or malignancies should undergo an esophagectomy [2, 6-7, 10].

## Conclusions

This case demonstrates a rare case of an upper GI bleed and acute respiratory obstruction from the regurgitation of food in a patient with a megaesophagus due to end-stage achalasia. Achalasia can predispose patients to develop pressure ulcers as food is regurgitated from a hypercontracted distal esophagus, enhancing friction and shear forces on the esophageal wall. Coupling this stress with damage to the mucosal protective barrier through gastric acid reflux, increased moisture from retained food, and ischemia can all play an essential role in the pathogenesis of pressure ulcer formation. The management of these ulcers is to treat the underlying cause. Although there are no curative treatments for achalasia, there are many surgical and medical therapies available that can provide patients symptomatic relief with an esophagectomy reserved for refractory cases or in patients who develop end-stage complications.
